# Activating newborn neurons suppresses depression and anxiety-like behaviors

**DOI:** 10.1038/s41467-019-11641-8

**Published:** 2019-08-21

**Authors:** Elif Tunc-Ozcan, Chian-Yu Peng, Yiwen Zhu, Sara R. Dunlop, Anis Contractor, John A. Kessler

**Affiliations:** 1 Department of Neurology, Feinberg School of Medicine, Northwestern University, Chicago, IL USA; 2 Department of Physiology, Feinberg School of Medicine, Northwestern University, Chicago, IL USA

**Keywords:** Neuroscience, Molecular medicine

## Abstract

The etiology of major depressive disorder (MDD), the leading cause of worldwide disability, is unknown. The neurogenic hypothesis proposes that MDD is linked to impairments of adult neurogenesis in the hippocampal dentate gyrus (DG), while the effects of antidepressants are mediated by increased neurogenesis. However, alterations in neurogenesis and endophenotypes are not always causally linked, and the relationship between increased neurogenesis and altered behavior is controversial. To address causality, we used chemogenetics in transgenic mice to selectively manipulate activity of newborn DG neurons. Suppressing excitability of newborn neurons without altering neurogenesis abolish the antidepressant effects of fluoxetine. Remarkably, activating these neurons is sufficient to alleviate depression-like behavior and reverse the adverse effects of unpredictable chronic mild stress. Our results demonstrate a direct causal relationship between newborn neuronal activity and affective behavior. Thus, strategies that target not only neurogenesis but also activity of newborn neurons may lead to more effective antidepressants.

## Abstract (web summary)

It is unclear if there is a causal link between increased neurogenesis and altered affective behaviors in major depressive disorders. Here, the authors show that selectively suppressing the excitability of newborn neurons, without altering neurogenesis, abolishes the antidepressant effects of fluoxetine.

## Introduction

The cellular and molecular mechanisms underlying the behavioral effects of antidepressants are not well defined. However, it is known that chronic antidepressant treatment prevents hippocampal volume reduction in major depressive disorder (MDD) patients and increases the production of new neurons in the subgranular neurogenic niche of the dentate gyrus (DG) of the hippocampus in preclinical models of MDD^1–7^. Indeed, decreased neurogenesis, structural abnormalities, and functional deficits are common in the hippocampus in MDD^5,8–12^ and are normalized by antidepressant treatments^5,7,13,14^, raising the possibility that adult-born neurons have a part in the depressive endophenotype. In line with this hypothesis, reducing the number of adult-born neurons by inhibiting cell proliferation with irradiation or genetic means leads to changes in affective behaviors in animal models^15^ and abolishes the effect of antidepressants^1,2,12,16^. Therefore, increased neurogenesis may be necessary for the effects of antidepressants, although this remains controversial because of the lack of specificity of the techniques used to target newborn neurons^17–19^. Further, it is unknown whether altering cell proliferation or the activity of the newly born neurons is sufficient by itself for an antidepressant-like effect.

The subgranular zone (SGZ) of the DG is the only region of the hippocampus where new neurons are continuously produced throughout life in all mammalian species thus far tested^20,21^. These adult-born neurons pass through multiple developmental stages before becoming functionally integrated into the hippocampal circuitry^22^. Electrophysiological recording of these neurons demonstrated that they receive synaptic inputs during 1–2 weeks after differentiation^23–25^. Also, after integration into the hippocampal circuity adult-born neurons are more excitable than mature granule neurons and display elevated long-term potentiation, which has been proposed to provide them with privileged roles in some forms of hippocampal dependent learning^26,27^. In addition, the adult-born neurons modulate the activity of developmentally born neurons within the DG, which influences the downstream circuitry to control behavior^28–30^. Recently, Anacker et al.^31^ demonstrated that adult-born neurons directly inhibit the activity of mature granule cells in the DG, which resulted in increased resilience against chronic stress^31^. Nevertheless, there has been a lack of direct evidence demonstrating that antidepressant effects are mediated by activity of newly generated DG neurons.

## Results

### Fluoxetine enhances behavior, neuronal number and activity

To define the involvement of neurogenesis and newborn neuronal activity in antidepressant action, first we treated C57Bl/6 mice with fluoxetine for 14 days and performed the tail suspension test (TST), the zero maze (ZM) and open-field tests (OFTs) to evaluate depression- and anxiety-like behaviors together with an analysis of locomotion (Supplementary Fig. 1). As expected, fluoxetine treated animals showed less immobility in the TST, spent more time in the open arms of the ZM, and also a greater proportion of distance in the center of the OFT chamber, indicating a reduction in depression- and anxiety-like behavioral phenotypes (Supplementary Fig. 1a–c). Additionally, there were no significant changes in the locomotor activity of these animals as assessed by the total distance traveled in the OFT (Supplementary Fig. 1d). Fluoxetine treatment also significantly increased the total number of dividing cells that are labeled with 5-bromo-2’-deoxyuridine (BrdU) or express Ki67 + in the SGZ of DG (Supplementary Fig. 1e–i), reproducing the well described effects of the antidepressants on neurogenesis^1,2,12,16,18,32^. We also examined expression of the immediate early gene c-Fos as a marker of neuronal activity. The number of c-Fos + cells was significantly increased in the DG after 14 days of fluoxetine treatment (Supplementary Fig. 1g, j). Taken together, these data indicate that fluoxetine treatment improves affective behavior in parallel with increases in neurogenesis and in neuronal activity in the DG.

### Effects of silencing newborn neurons on behavior

To investigate whether newly generated neurons are required for the antidepressant effects of fluoxetine, we used a chemogenetic approach in combination with transgenic mouse models^33^. The designer receptors exclusively activated by designer drugs (DREADDs), hM4Di and hM3Dq, are synthetic variants of human muscarinic receptors, coupled to Gi and Gq proteins, respectively^34,35^. They are exclusively activated by the exogenous ligand clozapine-N-oxide (CNO) with high efficacy^34^. First, we expressed the inhibitory DREADD, hM4Di, in newly generated granule neurons by crossing a transgenic mouse line with a tamoxifen-inducible Cre recombinase under the control of the *Ascl1* gene promoter^36^ to mice expressing STOP-floxed hM4Di (*Ascl1*-CreER^TM^;R26^LSL−hM4Di^ double-transgenic line referred to as + hM4Di mice, see Methods). + hM4Di mice express HA-Tagged hM4Di in Type 2 transit amplifying neural progenitor cells (NPCs) and their progeny, as well as a small subset of Type 1 neural stem cells (NSCs) after the administration of tamoxifen^37,38^. Upon the binding of CNO, hM4Di induces the canonical Gi pathway resulting in hyperpolarization of the neurons^35^. This provided a way of specifically and exclusively suppressing excitability of newborn neurons after they have integrated into the hippocampal circuitry. We have already shown the specificity of *Ascl1*-CreER^TM^ line, where high levels of recombination are seen in the SGZ of the DG^32,36^. To address the recombination efficacy of the hM4Di transgenes, we immunohistochemically analyzed HA-Tag expression in NPCs marked by SRY-box 2 (Sox2) and neuroblasts marked by doublecortin (Dcx) 2 days after 5 days of tamoxifen injection in adult animals. 69.4% ± 1.93% of HA-Tag + cells were colabeled with Sox2, while 27.4% ± 1.93% colocalized with Dcx, which showed that nearly all of HA-Tag-positive cells are initially precursors to the DG granule neurons. Subsequently, we examined expression of HA-Tagged hM4Di in the DG immunohistochemically after the recombined cells were allowed to mature for 21 days (Supplementary Fig. 2a) and found selective colocalization of the HA-Tag with granule cell markers. 86.6% ± 3.6% of the HA-Tag + cells were detected in NeuN + neurons in the granule cell layer, while only 2% ± 0.9 of them expressed the mature granule cell marker Calbindin, indicating that the majority of hM4Di + recombined cells were young granule neurons 3 weeks after tamoxifen injection (Supplementary Fig. 2b). Next, we confirmed CNO’s effect on newly generated DG neurons by performing patch clamp recordings from ±hM4Di neurons, which express YFP in the presence of hM4Di. We found that CNO decreased the number of action potentials elicited by depolarizing current injection in these neurons (Supplementary Fig. 2c, d), demonstrating that CNO mediated activation of hM4Di had a potent effect on the excitability of the neurons^39^. Additionally, expression of hM4Di without CNO treatment or exposing wild type adult-born DG neurons to CNO did not affect action potential firing (Supplementary Fig. 2c, d), which confirmed that both the hM4Di receptor and CNO are inert elements by themselves.

**Fig. 1:**
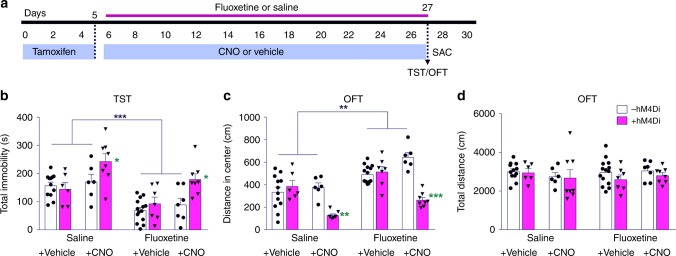
Silencing newborn dentate gyrus neurons prevents the effects of fluoxetine and alters baseline affective behavior. **a** Timeline representing the experimental design for determining whether silencing of newborn neurons inhibits the effects of fluoxetine treatment in Ascl1-CreER^TM^;R26^LSL−hM4Di^ mice. **b** Total time-spent immobile in tail suspension test, a measure of depression-like behavior (Fluoxetine *F*_1,60_ = 23.11, ***P *<* 0.0001; hM4Di *F*_1,60_ = 6.92, *P = 0.011; CNO *F*_1,60_ = 15.60, ***P < 0.0001; hM4Di*CNO *F*_1,60_ = 5.08, *P *=* 0.028; Tukey posthoc test + *hM4Di* *+* *CNO Saline* treatment versus other saline treatment groups *P < 0.05; + *hM4Di* *+* *CNO Fluoxetine* treatment versus other fluoxetine treatment groups *P < 0.05). **c** Distance spent in center exploration in open-field test, a measure of anxiety-like behavior (Fluoxetine *F*_1,60_ = 42.32, ***P *<* 0.0001; hM4Di *F*_1,60_ = 28.59, ***P *<* 0.0001; CNO *F*_1,60_ = 5.27, *P = 0.025; hM4Di*CNO *F*_1,60_ *=* 44.46, ***P *<* 0.0001; Tukey posthoc test + *hM4Di* *+* *CNO Saline* treatment versus other saline treatment groups **P < 0.01; + *hM4Di* *+* *CNO Fluoxetine* treatment versus other fluoxetine treatment groups ***P < 0.0001). **d** Total distance in open-field test, measure of locomotor activity (Fluoxetine *F*_1,60_ = 0.002, *P* *=* 0.965; hM4Di *F*_1,60_ = 1.297, *P* *=* 0.259; CNO *F*_1,60_ = 0.138, *P* = 0.711; hM4Di*CNO *F*_1,60_ = 0.030, *P* *=* 0.862). Data are presented as means ± s.e.m. and analyzed by three-way ANOVA

We then examined whether chronic chemogenetic in vivo inhibition of newly generated neurons alters the behavioral effects of fluoxetine. Transgenic mice received 3 weeks of daily intraperitoneal injection of fluoxetine or saline with or without chronic CNO supplementation in the drinking water (Fig. 1a). Inhibiting the activity of newborn neurons by CNO administration blocked the ameliorative effects of fluoxetine on affective behavior (Fig. 1b, c). Specifically, fluoxetine treatment decreased immobility in the TST in the absence of both hM4Di (-hM4Di) and CNO. This effect was reversed by CNO treatment to mice expressing hM4Di ( + hM4Di) (Fig. 1b). Interestingly, inhibiting activity of newborn neurons in the absence of fluoxetine ( + hM4Di + CNO in saline treated mice) increased immobility in the TST, indicating that the baseline activity of new neurons regulates behavior of mice in this test. Next, anxiety-like behavior was evaluated by the measurement of the distance traveled in the center of the OFT chamber (Fig. 1c). The findings paralleled the results with the TST. Fluoxetine treated −hM4Di and + hM4Di (−CNO) animals preferred to explore the center of the arena significantly more than saline treated animals, indicative of the anti-anxiety effects of fluoxetine. CNO treatment of mice expressing hM4Di blocked the anti-anxiety effects of fluoxetine, as indicated by the reduction in distance traveled in the center of the testing chamber compared to the other fluoxetine treated animals. Further, CNO treatment of + hM4Di animals in the absence of fluoxetine reduced the distance traveled in the center of the chamber, providing additional evidence that the baseline activity of new neurons regulates behavior even in the absence of fluoxetine. There were no significant differences among groups in terms of locomotor activity (Fig. 1d).

**Fig. 2:**
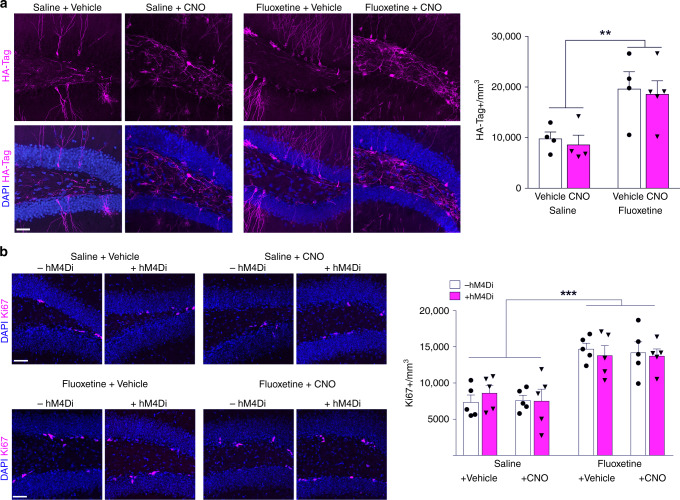
CNO silencing does not alter the increase in neurogenesis after fluoxetine treatment. **a** Representative images and quantification of HA-Tag + dentate gyrus cells (By two-way ANOVA: Fluoxetine *F*_1,13_ = 16.15, **P *=* 0.0015; CNO *F*_1,13_ = 0.2021, *P* = 0.660; Interaction *F*_1,13_ = 0.0009, *P* *=* 0.976). **b** Representative images and quantification of ki67-positive dentate gyrus cells (by three-way ANOVA: Fluoxetine *F*_1,32_ = 59.52, ***P *<* 0.0001; hM4Di *F*_1,32_ = 0.130, *P* = 0.721; CNO *F*_1,32_ = 0.143, *P* = 0.708; hM4Di*CNO *F*_1,32_ = 0.001, *P* *=* 0.980). Data are presented as means ± s.e.m. Scale bars 50 μm

When we analyzed the distribution of the HA-Tagged hM4Di + cells in the DG, we found that fluoxetine treatment increased the number of HA-Tag + cells regardless of CNO treatment (Fig. 2a), however it did not have a significant effect on the maturation of these adult-born neurons; as assessed by double labeling of HA-Tag + cell with neuronal maturity markers, Sox2 (Saline: 3.71% and Fluoxetine: 3.45%), Dcx (Saline: 16.24% and Fluoxetine: 16.81%), NeuN (Saline: 77.73% and Fluoxetine: 76.51%), and Calbindin (Saline: 2.32% and Fluoxetine: 3.23%) (Supplementary Fig. 3). As expected, fluoxetine increased neurogenesis measured by an increased number of Ki67 + cells in the DG (Fig. 2b). Neither hM4Di nor CNO alone or together altered the level of neurogenesis (Fig. 2b) and neuronal maturation (Supplementary Fig. 3).

**Fig. 3:**
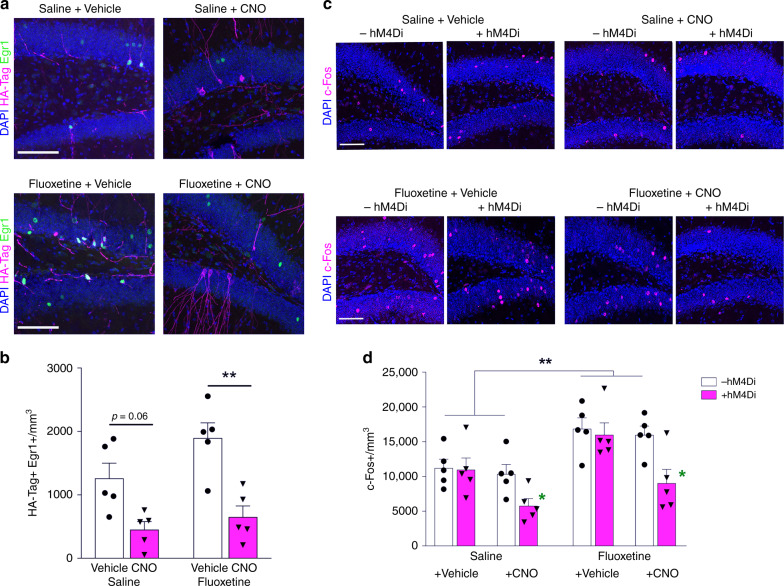
CNO prevents the fluoxetine-induced increase in activity of dentate gyrus neurons in vivo. **a** Representative images of HA-Tag + Egr1 + double-labeled dentate gyrus cells. **b** Quantification of HA-Tag + Egr1 + cells in the dentate gyrus (by two-way ANOVA: Fluoxetine *F*_1,16_ = 4.288, *P* *=* 0.0549; CNO *F*_1,16_ = 26.16, ***P = 0.0001; Interaction *F*_1,16_ = 1.184, *P* *=* 0.293). **c** Representative images of c-Fos-positive dentate gyrus cells. **d** Quantification of c-Fos expression in the dentate gyrus. (by three-way ANOVA: Fluoxetine *F*_1,32_ = 21.55, ***P *<* 0.0001; hM4Di *F*_1,32_ = 9.01, **P *=* 0.005; CNO *F*_1,32_ = 10.85, **P = 0.002; hM4Di*CNO *F*_1,32_ = 6.14, *P *=* 0.019; Tukey posthoc test + *hM4Di* *+* *CNO Saline* treatment versus other saline treatment groups *P < 0.05; + *hM4Di* *+* *CNO Fluoxetine* treatment versus other fluoxetine treatment groups *P < 0.05). Data are presented as means ± s.e.m. Scale bars 50 μm

To verify silencing effects of CNO on activity of newborn neurons in vivo, we examined the expression of immediate early genes Early Growth Response 1 (Egr1) and c-Fos, which are upregulated by neuronal activity^40^ (Fig. 3). Fluoxetine treatment increased the number of HA-Tag + Egr1 + double-labeled cells in the DG indicating an increase in newborn neuron activity, while CNO administration suppressed newborn neuronal activity in both Saline and Fluoxetine groups, as measured by decreased total of HA-Tag + Egr1 + cells (Fig. 3a, b). These findings confirmed that the inhibition of activity observed in CNO treated + hM4Di neurons in vitro (Supplementary Fig. 2) also occurred in vivo. Similarly, fluoxetine treatment increased the number of c-Fos immunoreactive cells in the DG with a representation of a more global increase in the DG activity (Fig. 3c, d). However, CNO treatment of + hM4Di mice abolished this effect of fluoxetine on c-Fos expression. CNO treatment of + hM4Di animals in the absence of fluoxetine also reduced the number of c-Fos + cells below baseline levels (Fig. 3c, d), correlating with the behavioral findings in the TST and OFT (Fig. 1), while CNO treatment of -hM4Di mice had no effect. Taken together, these findings indicate that the activity of newly generated neurons in the DG exerts profound effects on behavior. Further, activity of newly generated neurons is necessary for the behavioral effects of fluoxetine. While many prior studies have demonstrated the parallel effects of antidepressants on neurogenesis and behavior, our findings demonstrate that there is a causal relationship between the increased number and activity of newly generated neurons and the behavioral phenotypes.

### Activating newborn neurons leads to antidepressant effect

**Fig. 4:**
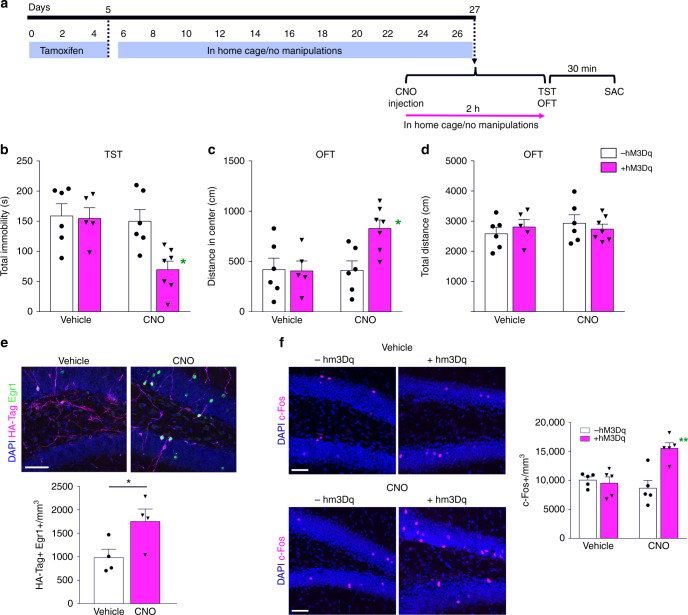
Acute activation of newborn dentate gyrus neurons results in antidepressive effects. **a** Timeline representing experimental design for determining whether enhancing the activity of newborn neurons induces an antidepressant effect in Ascl1-CreER^TM^;R26^LSL−hM3Dq^ mice. **b** Total time spent immobile in tail suspension test, a measure of depression-like behavior (hM3Dq *F*_1,20_ = 5.86, *P = 0.025; CNO *F*_1,20_ = 7.16, *P = 0.015; Interaction *F*_1,20_ = 4.72, *P = 0.042; Tukey posthoc test + *hM3Dq* *+* *CNO* versus other groups *P < 0.05). **c** Center exploration distance in open-field test, a measure of anxiety-like behavior (hM3Dq *F*_1,20_ = 4.42, *P = 0.048; CNO *F*_1,20_ = 4.65, *P = 0.044; Interaction *F*_1,20_ = 4.98, *P = 0.037; Tukey posthoc test + *hM3Dq* *+* *CNO* versus other groups *P < 0.05). **d** Total distance in open-field test, measure of locomotor activity (hM3Dq *F*_1,20_ = 0.005, *P* = 0.945; CNO *F*_1,20_ = 0.418, *P* = 0.525; Interaction *F*_1,20_ = 0.927, *P* = 0.347). **e** Representative images and quantification of HA-Tag + Egr1 + double-positive cells in the dentate gyrus (*t*_6 = _2.459, *P = 0.049). **f** Representative images and quantification of c-Fos expression in the dentate gyrus (hM3Dq *F*_1,16_ = 10.11, **P = 0.006; CNO *F*_1,16_ = 5.31, *P = 0.035; Interaction *F*_1,16_ = 13.79, **P = 0.002; Tukey posthoc test + *hM3Dq* *+* *CNO* versus other groups **P < 0.01). Data are presented as means ± s.e.m. and analyzed by two-way ANOVA (**b**, **c**, **d**, and **f**) or two-tailed Student’s *t*-test (**e**). Scale bar 50 μm

The findings described above demonstrated that the baseline level of activity of newly generated neurons regulates behavior. Next, we asked whether enhancing excitability of newly generated neurons without an increase in newborn neuron numbers would be sufficient to induce effects similar to those associated with fluoxetine treatment and increased neurogenesis. To explore this, we produced an Ascl1-CreER^TM^;R26^LSL−hM3Dq^ double-transgenic line (referred as + hM3Dq mice), where HA-Tagged hM3Dq is expressed in NPCs and their progeny after administration of tamoxifen, similar to hM4Di expression. Upon the binding of CNO, hM3Dq induces the canonical Gq pathway resulting in increased firing of the neurons^35^. Three weeks after tamoxifen induced recombination, we tested the effects of acute activation of adult-born DG neurons by injecting CNO 2 h before behavioral testing (Fig. 4a), as plasma levels of CNO peak within 30 min and decline over the following 2 h^41^. Acute activation of newly generated adult DG neurons with CNO led to behavioral phenotypes similar to fluoxetine treatment. + hM3Dq CNO treated animals showed decreased total immobility in TST compared to Vehicle and –hM3Dq CNO groups (Fig. 4b), representing a reduction in depression-like behavior. At the same time, these animals were less anxious as measured by a significantly increased exploration in the center of OFT chamber (Fig. 4c). No significant differences were found in locomotor activity, assessed by OFT total distance (Fig. 4d). At the cellular level, acute hM3Dq activation significantly increased numbers of HA-Tag + Egr1 + double-labeled newborn granule neurons (Fig. 4e) and c-Fos + neurons globally in the DG (Fig. 4f). The number of newborn neurons and cell proliferation, as measured by the number of HA-Tag + and Ki67 + cells in the DG (Supplementary Fig. 5a, b) were not changed by either hM3Dq expression alone or by acute activation of hM3Dq expressing neurons by CNO. Thus, enhancing the excitability of small cohort of newborn DG granule neurons in the absence of changes in hippocampal neurogenesis leads to an antidepressant effect, further substantiating the role of newly generated neurons in regulating depression- and anxiety-like behaviors.

**Fig. 5:**
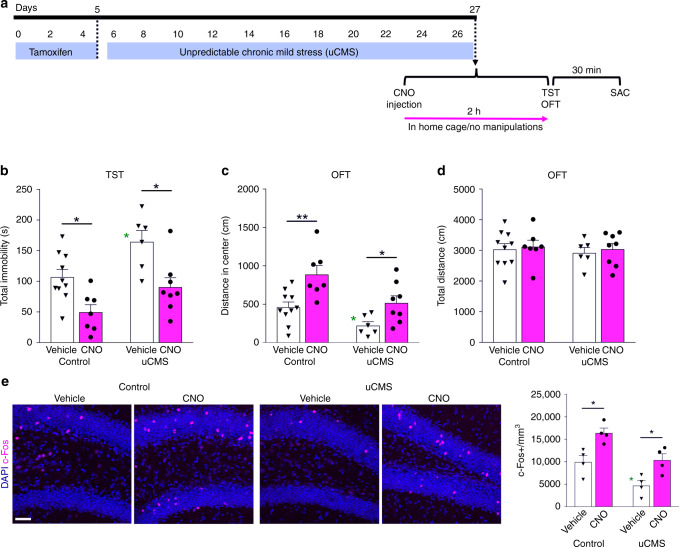
Acute activation of newborn dentate gyrus neurons reverses the behavioral effects of unpredictable chronic mild stress (uCMS). **a** Timeline representing the experimental design for determining whether acutely enhancing the activity of newborn neurons in adult hippocampus reverses the behavioral effects of unpredictable chronic mild stress (uCMS) in Ascl1-CreER^TM^;R26^LSL−hM3Dq^ mice. **b** Total time-spent immobile in tail suspension test, a measure of depression-like behavior (uCMS *F*_1,27_ = 11.23, **P = 0.002; CNO *F*_1,27_ = 19.93, ***P = 0.0001; Interaction *F*_1,27_ = 0.313, *P* = 0.581; Tukey posthoc test *Control* *+* *Vehicle* versus *uCMS* *+* *Vehicle* *P < 0.05). **c** Center exploration distance in open-field test, a measure of anxiety-like behavior (uCMS *F*_1,27_ = 12._1,_ **P = 0.002; CNO *F*_1,27_ = 17.04, ***P = 0.0003; Interaction *F*_1,27_ = 0.564, *P* = 0.459; Tukey posthoc test *Control* *+* *Vehicle* versus *uCMS* *+* *Vehicle* *P < 0.05). **d** Total distance in open-field test, measure of locomotor activity (uCMS *F*_1,27_ = 0.242, *P* = 0.627; CNO *F*_1,27_ = 0.267, *P* = 0.609; Interaction *F*_1,27_ = 0.008, *P* = 0.930). **e** Representative images and quantification of c-Fos expression in the dentate gyrus (uCMS *F*_1,12_ = 19.19, ***P = 0.0009; CNO *F*_1,12_ = 22.01, ***P = 0.0005; Interaction *F*_1,12_ = 0.088, *P* = 0.771; Tukey posthoc test *Control* *+* *Vehicle* versus *uCMS* *+* *Vehicle* *P < 0.05). Data are presented as means ± s.e.m. and analyzed by two-way ANOVA. Scale bar 50 μm

We tested whether increasing newborn neuronal activity is also sufficient to avert the adverse effects of unpredictable chronic mild stress (uCMS), which is known to elicit behavioral phenotypes associated with MDD that are reversed by fluoxetine treatment^42^. Ascl1-CreER^TM^;R26^LSL−hM3Dq^ ( + hM3Dq) mice were exposed to 3 weeks of uCMS (Supplementary Table 1) and then evaluated behaviorally with or without acute CNO treatment to assess the effects of excitation of newborn neurons via hM3Dq receptor activation (Fig. 5a). Exposure to stress produced robust depression-like (Fig. 5b) and anxiety-like (Fig. 5c) behaviors as measured by increased total immobility in the TST and decreased center distance in the OFT, respectively, compared to the control vehicle group. By contrast, CNO-treated uCMS mice exhibited control levels of total immobility in the TST and center exploration in the OFT (Fig. 5b, c), indicating that increased newborn neuronal activity is sufficient to promote stress resilience. There were no locomotor activity differences among the experimental groups in the OFT (Fig. 5d). Parallel to behavioral changes, CNO administration significantly increased the number of c-Fos + cells in the DG in both control and uCMS conditions (Fig. 5e), though there was a significant reduction of c-Fos expression in uCMS exposed animals compared to controls. We also quantified the number of HA-Tag + cells in the DG and found that neither uCMS nor acute CNO treatment changed the numbers of HA-Tagged hM3Dq expressing cells (Supplementary Fig. 5), further validating the influence of newborn neuronal activity on behavioral phenotypes without changes of the number of neurons. The maturation of these newborn neurons was also not affected by these variables as assessed by double labeling of HA-Tag + cell with neuronal maturity markers; Sox2, Dcx, NeuN, and Calbindin (Supplementary Fig. 6). Thus, increased activation of newly generated neurons is sufficient to promote stress resilience.

Our results demonstrate that activity of adult-born neurons in the DG of the hippocampus is both necessary and sufficient for antidepressant action of fluoxetine. Cell autonomous restriction of our experimental manipulations, without drug or radiation induced cell death^43^, and activating and silencing DREADD receptors specific to the population of newly generated neurons enabled us to establish this causal relationship. The pathophysiology of depression and anxiety is still poorly understood, and current medications are almost exclusively based on modifications of first-generation antidepressants, which were developed decades ago. Almost a third of patients fail to respond to any of the currently available medications, and treatment with these medications is associated with many significant complications and side effects^44^. Our results suggest that strategies that target both neurogenesis and activity of newborn neurons may lead to more effective antidepressants.

## Methods

### Animals

All animal procedures were approved by the Northwestern University Institutional Animal Care and Use Committee (IACUC). All experiments were performed in accordance with the Public Health Service Policy on Humane Care and Use of Laboratory Animals. All animals were housed 3–5 per cage on a 14:10 h light:dark cycle in a controlled environment and received food and water ad libitum. All behavioral testing was conducted during the light period.

Eight to 10-week-old, naive C57Bl/6 male and female mice (Charles River, Wilmington, MA, USA) were used for antidepressant (fluoxetine) experiments (Supplementary Fig. 1), where animals were randomly assigned to experimental groups, saline or fluoxetine treatment, for 14 days.

hM4Di and hM3Dq floxed mice (hM4Di^−/+^ - Stock#026219 and hM3Dq^−/+^- Stock#026220)^33^ were purchased from Jackson Laboratories (Bar Harbor, ME, USA) and were mated to mice containing tamoxifen-inducible Cre recombinase under the control of the *Ascl1* promoter (Ascl1-CreER^TM^)^32,36,37^ to produce double-transgenic progeny, Ascl1-CreER^TM^;R26^LSL−hM4Di^ ( + hM4Di), and Ascl1-CreER^TM^;R26^LSL−hM3Dq^ ( + hM3Dq). Mice were genotyped through PCR using genomic DNA and Jackson Laboratories-provided primers. Tamoxifen-induced expression of hM4Di and hM3Dq was performed at 8–10-weeks of age. Tamoxifen administration led to conditional expression of HA-tagged hM4Di and hM3Dq, and also mCitrine yellow fluorescent protein (YFP) in *Ascl1*-expressing cells, which was used as the fluorophore for electrophysiological examination. Cre and/or DREADD-negative littermates from heterozygote breeding were used as controls. At least three separate cohorts of mice were run for all experiments.

### Drug administration

Fluoxetine (Sigma Aldrich, St. Louis, MO, USA) was dissolved in saline and administered at a dose of 10 mg kg^−1^ via intraperitoneal (i.p.) injection daily for 14 or 21 consecutive days. Control animals received daily i.p. injections of saline for 14 or 21 consecutive days accordingly. Bromodeoxyuridine (BrdU, Sigma Aldrich) was dissolved in Phosphate Buffer Saline (PBS) and for neuronal labeling mice received four consecutive i.p. injections of 50 mg kg^−1^ BrdU at 2 h intervals and were sacrificed 24 h after the last injection. For conditional expression of hM4Di and hM3Dq tamoxifen (Sigma Aldrich) was dissolved in 100% ethanol, suspended in corn oil and administered via i.p. injection at 180 mg kg^−1^ for 5 consecutive days. Water soluble Clozapine-N-oxide (CNO) was purchased from Hello Bio (Princeton, NJ, USA). For acute treatment, animals received i.p. injection of 2 mg kg^−1^ CNO 2 h before the behavioral tests. Control animals received i.p. saline injections in the same time course. For chronic treatment, CNO was dissolved in sterile drinking water at a concentration of 0.25 mg ml^−1^. At this concentration, the mice received approximately 5 mg kg^−1^ per day CNO, as they weigh 30 g and consume 6 ml water per day (preliminary data). Animals had this CNO solution as their only source of drinking water ad libitum until they were sacrificed for sample collection. Freshly prepared CNO solution was replenished as needed every 2–4 day for the duration of chronic treatment. Vehicle chronic treatment consisted of regular drinking water.

### Hippocampal slice electrophysiology

Mice were anesthetized with isoflurane followed by ketamine/xylazine, then rapid cardiac perfusion was performed using ice-cold sucrose artificial cerebrospinal fluid (ACSF) containing the following (in mM): 85 NaCl, 2.5 KCl, 1.25 NaH_2_PO_4_, 25 NaHCO_3_, 25 glucose, 75 sucrose, 0.5 CaCl_2_, and 4 MgCl_2_, equilibrated with 95% O_2_/5% CO_2_ before mice were decapitated. Brains were rapidly removed and placed in the same ice-cold sucrose ACSF as described above. Coronal hippocampal slices (250 μm thick) were prepared using a Leica 1000S vibratome and transferred to a heated (28–32 °C) holding chamber containing the same sucrose ACSF, which was gradually exchanged for regular ACSF containing the following (in mM): 125 NaCl, 2.5 KCl, 1.25 NaH_2_PO_4_, 25 NaHCO_3_, 25 glucose, 2 CaCl_2_, and 1 MgCl_2_, equilibrated with 95% O_2_/5% CO_2_ at room temperature. After being incubated with regular ACSF for at least 1 h, slices were transferred to a recording chamber and perfused continuously with oxygenated regular ACSF at a flow rate of 2 ml min^−1^. Hippocampal dentate granule neurons were visualized under differential interference contrast optics and hM4Di expressing neurons were identified based on YFP reporter fluorescence. Recording electrodes were made from borosilicate glass pipettes and had a resistance of 3.5–6.5 MΩ when filled with an internal solution containing the following (in mM): 130 K-gluconate, 10 KCl, 10 HEPES, 4 Mg-ATP, 0.3 Na-GTP, and 10 Na_2_Phosphocreatine, pH adjusted to 7.3 with KOH. Input resistances were determined in whole-cell voltage-clamp mode by holding cells at −70 mV and presenting a −5 mV hyperpolarizing voltage step with a duration of 100 ms. Input–output (I/O) relationship was generated by injecting various amount of current to hold cell membrane potential close to −70 mV while further injecting 0–100 pA current in 10 pA increments and counting spike number in response to each current step. CNO (10 μm) was bath applied for at least 3 min before any measurements and was maintained in the bath throughout the rest of each recording. All data were acquired at 10 kHz at room temperature and analyzed using pClamp 10 software (Molecular Devices, San Jose, CA, USA).

### Unpredictable chronic mild stress (uCMS)

Mice were subjected to various unpredictable chronic mild stressors for 3 weeks. Before the start of any stressful stimulus, animals were transferred to a clean room used for uCMS manipulations and exposed to 1 or 2 of the stressors listed below each day (Supplementary Table 1). Stressors were performed on a randomized schedule, and each stress was administered for a minimum of 2 h, except light cycle disturbances and restraint stress. At the end of each daily stress period, animals were replaced into clean cages and returned to the housing facility. Control animals were handled only for cage changes, behavioral tests and CNO or vehicle injection.

1. Wet bedding: Home cage bedding was dampened by pouring ~500 ml of clean water, which would be enough for not to cause pooling of water.
2. No bedding: All bedding from each home cage was removed.
3. Tilted cage: Home cage was tilted ~45° by using a sturdy object that would remain in place as the animal moves around.
4. Light cycle disturbances: Animals were exposed to regular room light during the night period or regular room light was off during day time.
5. Social stress: Mice were transferred from their home cage to the cage of a neighboring mice that has been removed for 3 h.
6. No bedding + water: All bedding from each home cage was removed and water (warmer than room temperature ~30 °C) was added a depth of ~ mm. Animals were towel dried prior to placement into clean cages.
7. Restraint stress: Mice were placed in a 50-ml plastic Falcon tube with openings in both sides for breathing for 1 h.
8. Predator smell: A filter paper soaked with 5 μl of 10% 2,4,5-Trimethylthiazoline (a component of fox feces and the most commonly used synthesizable reagent for inducing innate fear in rodents) was placed into the home cage.
9. No bedding + tilted cage: After removing the bedding, each home cage was tilted ~45° by using a sturdy object that would remain in place as the animal moves around.

### Behavioral analysis

For all behavioral analyses, mice were transferred to the testing room 1 h prior to testing for acclimation to the test environment. All behavioral apparatus was wiped with 70% ethanol prior to each trial and between trials. The zero maze (ZM) and open-field (OF) tests were performed at the Northwestern University Behavioral Phenotyping Core Facility. Behavioral analyses were performed by a single experimenter, blind to the experimental condition. All animals were sacrificed after the last behavioral test to collect the samples.

Zero maze (ZM): The ZM consisted of a 56 cm diameter round track divided into two closed quadrants separated by 180 degrees and two open quadrants separated by 180 degrees. The closed quadrants were enclosed by 15 cm high walls and the open quadrants had no walls. The maze was elevated 46 cm above the ground and testing was conducted under white light. The mouse was placed into a closed quadrant and allowed to move freely for 10 min with the activity being recorded and tracked by LimeLight 3 software (Actimetrics, Coulbourn Instruments, USA). Once the experiment completed, the software was used to produce the data for the percentage of time the mouse spent in open quadrants.

Open field (OF): The OF apparatus consisted of a 56 × 56 cm open arena with 30 cm high walls. The mouse was placed into the center of the arena and allowed to move freely for 5 min with the activity being recorded and tracked by LimeLight 3 software (Actimetrics, Coulbourn Instruments). The software recorded and analyzed total distance traveled in the inner (28 × 28 cm central area of the OF) and outer areas of the arena.

Tail suspension test (TST): Tape was affixed to the mouse’s tail 2 cm from the tip and the mouse was suspended from a horizontal bar at a height of 30 cm. Cylindrical plastic tubes were placed at the base of the tail to prevent tail climbing. The mouse was suspended for 6 min and video recordings of the test were quantified by an observer blinded to experimental condition. The total time spent in an immobile posture were measured.

### Immunohistochemistry

Mice were transcardially perfused with PBS followed by 4% paraformaldehyde. Brains were fixed overnight in 4% paraformaldehyde and then transferred to 30% sucrose for 24 h. Forty micrometer thick floating sections were obtained using a microtome (Microm HM 450, Thermo Fisher, Waltham, MA, USA). Tissue sections were washed three times in PBS, blocked in 10% normal serum with 0.25% Triton X-100 in PBS for 1 h at room temperature (RT), and incubated overnight at 4 °C in primary antibody diluted in 1% bovine serum albumin (BSA) with 0.25% Triton X-100 in PBS. Sections were washed three times in PBS and then incubated for 1 h at RT with a fluorophore-conjugated secondary antibody (Alexa-488, Alexa-555, or Alexa-647, Thermo Fisher) and 4′,6-diamidino-2-phenylindole(DAPI) for nuclear stain (Invitrogen, Carlsbad, CA, USA). Following three final washes in PBS, floating sections were mounted with ProLong Gold Antifade Reagent (Life Technologies, Carlsbad, CA, USA). Primary antibodies used were: rat anti-BrdU (1:250, ab6326, Abcam), chicken anti-Calbindin (1:1000, #CPCA, EnCor), rabbit anti-c-Fos (1:500, #26192–1-AP, Proteintech), guinea pig anti Doublecortin (1:500, AB2253, Millipore), rabbit anti-Egr1 (1:500, #4153, Cell Signaling), rabbit anti-HA-Tag (1:500, #3724, Cell Signaling), mouse anti-HA-Tag (1:500, MMS-101P, Biolegend), rabbit anti-Ki67 (1:500, ab15580, Abcam), mouse anti-NeuN (1:500, MAB377, Millipore), goat anti-Sox2 (1:500, sc-17320, Santa Cruz).

For BrdU detection, floating sections were incubated in 10 mM sodium citrate buffer with 0.05% Tween-20, pH 6.0 for 20 min at 95 °C, cooled to RT, washed three times in PBS, and transferred to 2 N HCl for 30 min at RT. Sections were then quenched in 0.1 M sodium tetraborate, pH 8.5 for 10 min at RT and washed in PBS. Blocking and antibody incubation were then performed as described above.

### Confocal imaging and quantification

Images were acquired using a Leica TCS SP5 Confocal Microscope. *Z*-stacks of the dentate gyrus were obtained (step size: 2 µm) using sequential scanning to prevent bleed-through between fluorophores. Six or more *Z*-stacks of equal thickness and equivalent rostrocaudal position were quantified for each sample. Stereological cell counting was performed using ImageJ software. For per volume quantification, cell counts were normalized to the volume of the dentate gyrus granule cell layer. All imaging and quantification were performed blinded to experimental condition.

### Statistical analysis

Statistical analyses were performed via two-tailed paired Student’s *t*-test, three-way ANOVA or two-way ANOVA with Tukey’s paired comparisons test for correction for multiple comparisons, as indicated in the figures and figure legends. SPSS and GraphPad Prism 7 software were used for the analyses. Normality was assessed using the Shapiro–Wilk and Kolmogorov–Smirnov tests and equality of variances was verified using the *F*-test of equality of variance. The significance threshold used was *P* < 0.05. The exact sample size for each experimental condition is represented in the figures as symbols. All data are reported as means ± s.e.m.

### Reporting summary

Further information on research design is available in the Nature Research Reporting Summary linked to this article.

## Supplementary information

Supplementary Information

Reporting Summary

## Data Availability

The data that support the findings of this study are available from the corresponding author upon reasonable request.
