# Author Correction: Activating newborn neurons suppresses depression and anxiety-like behaviors

**DOI:** 10.1038/s41467-025-66664-1

**Published:** 2025-12-10

**Authors:** Elif Tunc-Ozcan, Chian-Yu Peng, Yiwen Zhu, Sara R. Dunlop, Anis Contractor, John A. Kessler

**Affiliations:** 1 Department of Neurology, Feinberg School of Medicine, Northwestern University, Chicago, IL USA; 2 Department of Physiology, Feinberg School of Medicine, Northwestern University, Chicago, IL USA

Correction to: *Nature Communications* 10.1038/s41467-019-11641-8, published online 21 August 2019

In the version of the article initially published, in the “Drug administration” section of the Methods, the text “CNO was dissolved in sterile drinking water at a concentration of 0.25 mg ml^–1^” should have read “CNO was dissolved in sterile drinking water at a concentration of 0.025 mg ml^–1^”. This notice serves to amend the error.

