# Supplementary material for: Activating newborn neurons suppresses depression and anxiety-like behaviors
Source: Nat Commun. 2019 Aug 21;10:3768. doi: 10.1038/s41467-019-11641-8 (PMC6704083; doi:10.1038/s41467-019-11641-8)
Supplement: Supplementary file 1 — Supplementary Information [file 41467_2019_11641_MOESM1_ESM.pdf]

Supplementary information

Activating Newborn Neurons Suppresses Depression and Anxiety-like Behaviors

Tunc-Ozcan et al.

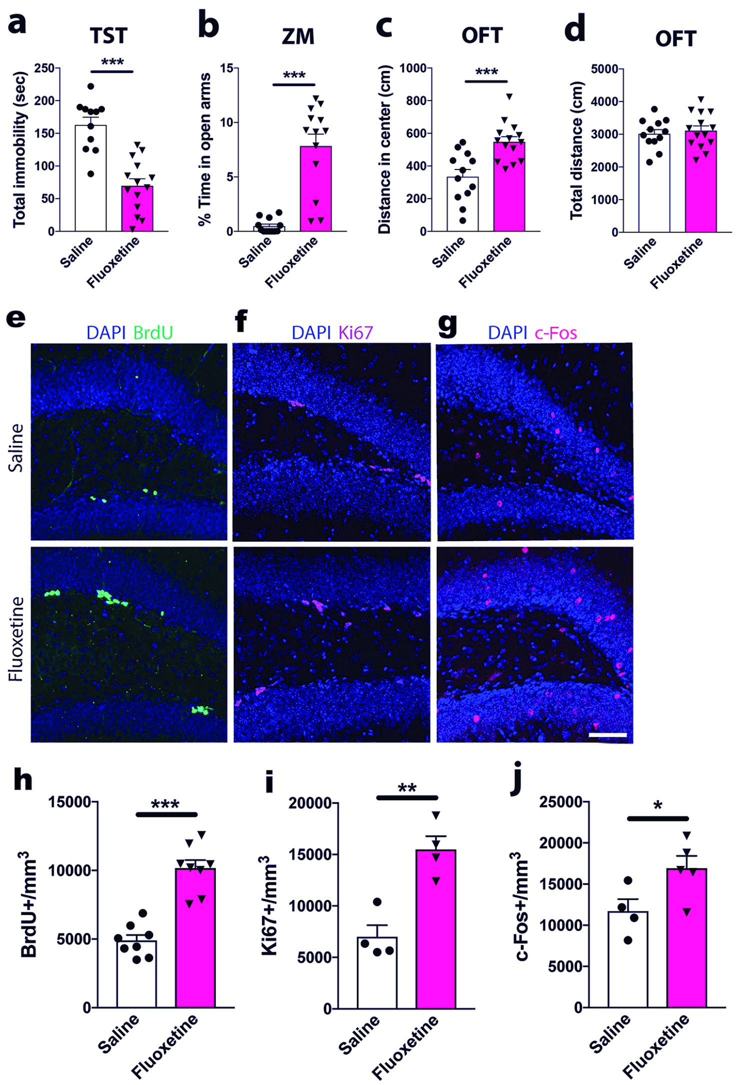

Supplementary Fig. 1: Fluoxetine treatment enhances behavior and increases number and activity of dentate gyrus neurons. a, Total time spent immobile in tail suspension test, a measure of depression-like behavior ($t_{23} = 5.81$, ***$P < 0.0001$). **b,** Percent time spent in the open arms of the zero maze, a measure of depression- and anxiety-like behaviors ($t_{23} = 6.34$, ***$P < 0.0001$). **c,** Distance spent in center exploration in open field test, a measure of anxiety-like behavior ($t_{24} = 3.97$, ***$P < 0.636$). **d,** Total distance in open field test, a measure of locomotor activity ($t_{24} = 0.48$, $P = 0.000$). **e-g,** Representative images showing expression of

BrdU, ki67 and c-Fos in the DG of the saline or fluoxetine treated animals. Scale bar 50 µm. **h,** Quantification of BrdU positive DG cells (t_{14} = 7.11, ***P < 0.0001). **i,** Quantification of ki67 positive DG cells (t_6 = 4.81, **P = 0.003). **j,** Quantification of c-Fos positive DG cells (t_7 = 2.40, *P = 0.05). Data presented as means ± s.e.m. and analyzed by two-tailed Student's *t*-test. One animal was excluded from tail suspension test analysis due to unstable fix during suspension, and one animal was excluded from the zero maze test analysis due to jumping out from the maze during the test.

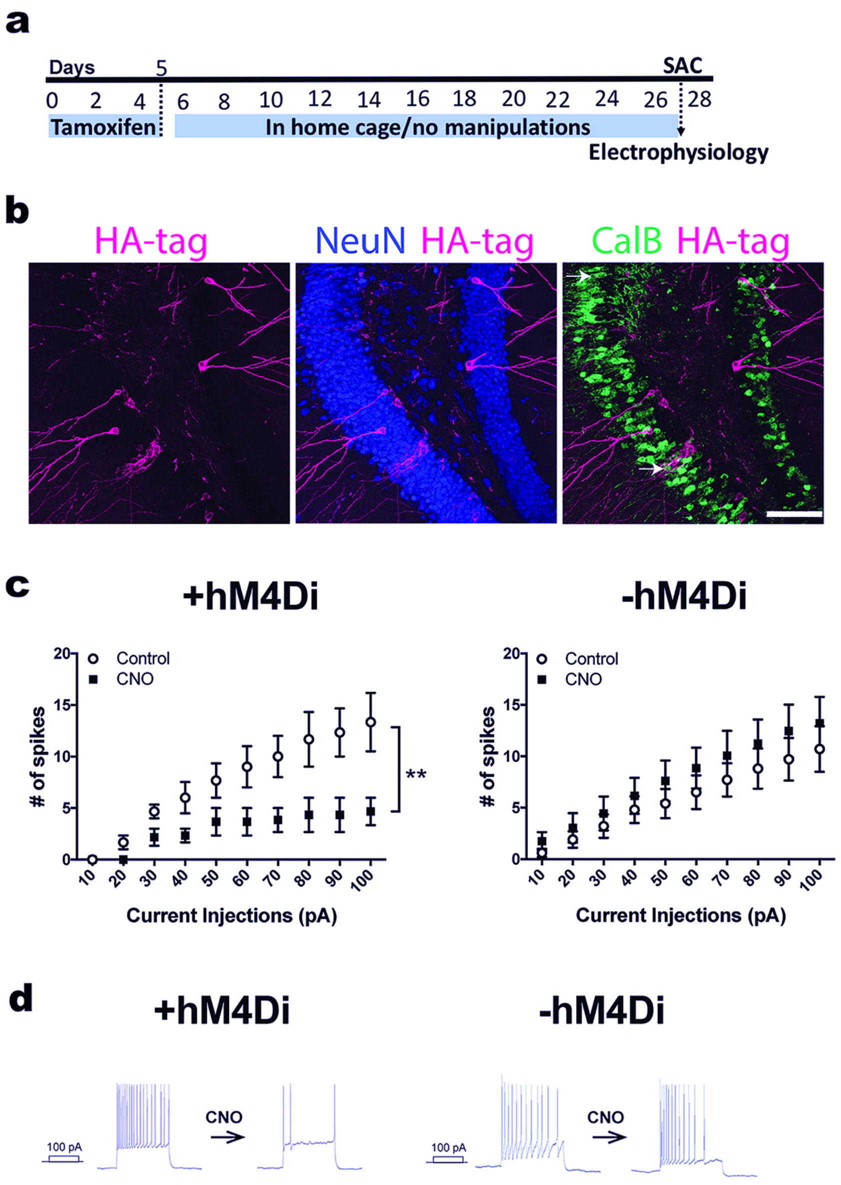

Supplementary Fig. 2: CNO decreases neuronal excitability of adult-born dentate gyrus neurons in Ascl1-CreER^{TM};R26$^{LSL\text{-}hM4Di}$ double transgenic mice (+hM4Di). a, Timeline representing experimental design to conditionally express hM4Di in the newborn neurons of the adult dentate gyrus to determine silencing effects of CNO in hippocampal slice electrophysiology. **b,** Representative confocal microscopy images showing selective expression of HA-tagged hM4Di in the dentate gyrus and colocalization with NeuN and Calbindin (CalB,

white arrows). **c,** The mean number of spikes during depolarizing current steps in the presence or absence of 10μM CNO (+hM4Di: Current injection*CNO $F_{9,18}$ = 3.607, P = 0.009, n = 3 cells/3 mice; -hM4Di: Current injection*CNO $F_{9,36}$ = 1.1, P = 0.387, n = 5 cells/4 mice). Data presented as means ± s.e.m. and analyzed by repeated measures two-way ANOVA. **d,** Representative traces of action potentials elicited by current injection from whole-cell current-clamp electrophysiological recordings of the dentate gyrus neurons before and after 10 min bath application of 10μM CNO.

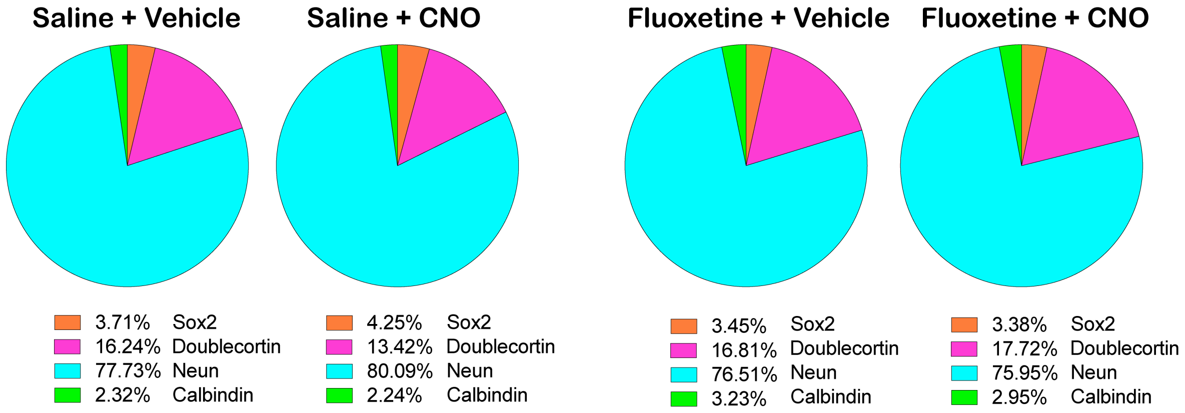

Supplementary Fig. 3: Fluoxetine and/or CNO supplementation does not change the maturation of dentate gyrus newborn neurons in Ascl1-CreER^{TM};R26$^{LSL\text{-}hM4Di}$ mice after 3 weeks of treatment. Immunohistochemical analyses of double labelling of HA-Tagged hM4Di cells with markers of progenitors of the granule neurons and neuronal maturity; Sox2, Doublecortin, NeuN and Calbindin. Data presented as percent of total HA-Tag+ cells in the dentate gyrus.

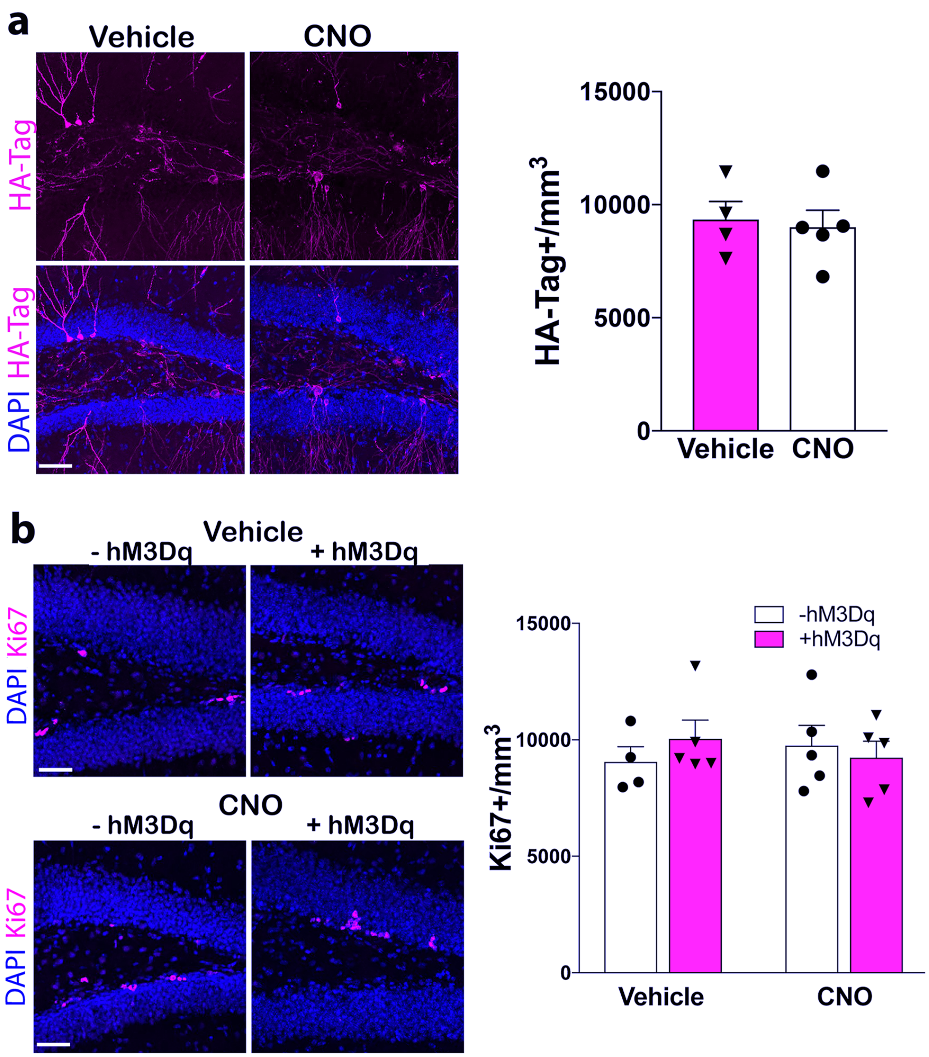

Supplementary Fig. 4: Acute activation of adult-born dentate gyrus neurons does not alter the number of newborn neurons in the dentate gyrus. a, Representative images and quantification of HA-tagged hM3Dq expression in the dentate gyrus (by two-tailed Student's *t*-test: t_7 = 0.2996, P = 0.773). **b,** Representative images and quantification of ki67 expression in the dentate gyrus (by two-way ANOVA: hM3Dq $F_{1,15}$ = 0.092, *P* = 0.766; CNO $F_{1,15}$ = 0.006, *P* = 0.940; Interaction $F_{1,15}$ = 0.931, *P* = 0.350). Data are presented as means ± s.e.m. Scale bar 50 µm.

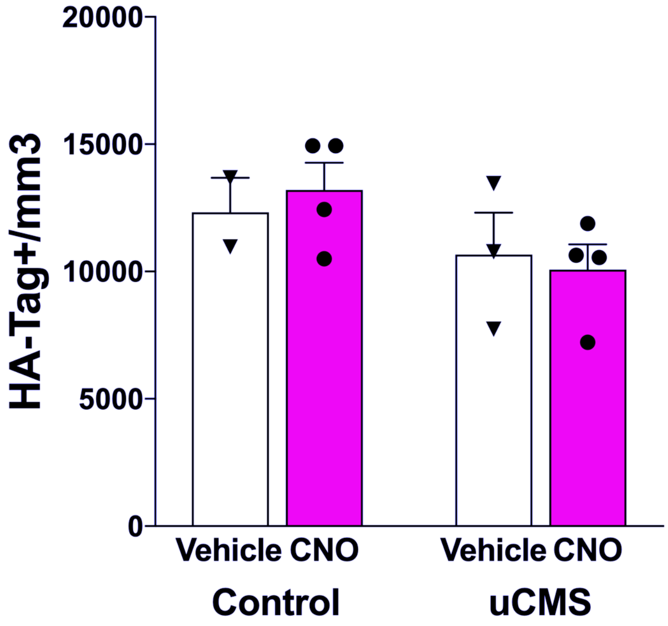

Supplementary Fig. 5: Three weeks of unpredictable chronic mild stress (uCMS) and/or acute CNO administration does not alter the number of HA-tagged newborn neurons in the dentate gyrus. Quantification of HA-tag+ cells in the dentate gyrus (uCMS $F_{1,9}$ = 3.368, P = 0.0997; CNO $F_{1,9}$ = 0.0129, P = 0.912; Interaction $F_{1,9}$ = 0.316, P = 0.588). Data are presented as means ± s.e.m. and analyzed by two-way ANOVA.

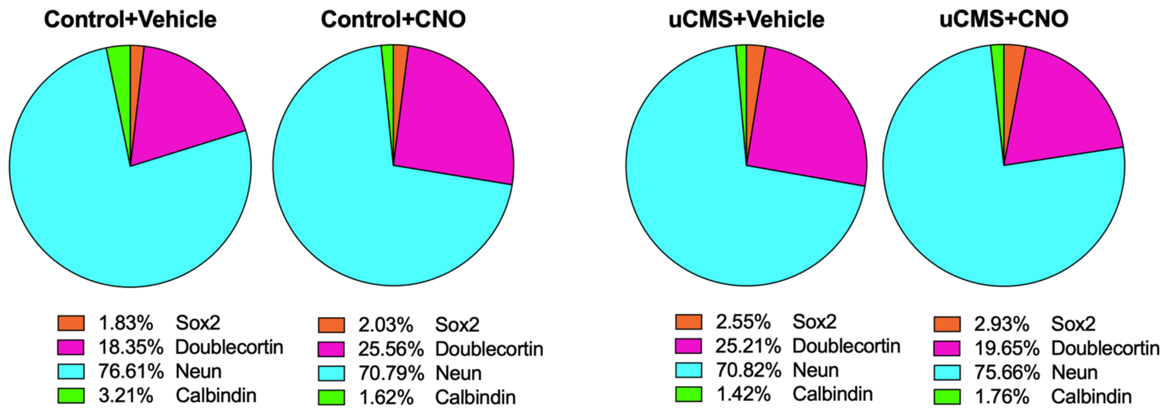

Supplementary Fig. 6: Three weeks of unpredictable chronic mild stress (uCMS) and/or acute CNO administration does not change the maturation of dentate gyrus newborn neurons in Ascl1-CreER^{TM};R26$^{LSL\text{-}hM3Dq}$ mice. Immunohistochemical analyses of double labelling of HA-Tagged hM3Dq cells with markers of progenitors of the granule neurons and neuronal maturity; Sox2, Doublecortin, NeuN and Calbindin. Data presented as percent of total HA-Tag+ cells in the dentate gyrus.

Supplemental Table 1: Experimental Stressors and Scheduling for Unpredictable Chronic Mild Stress (uCMS)							
	**Day 1**	**Day 2**	**Day 3**	**Day 4**	**Day 5**	**Day 6**	**Day 7**
**Week 1**	Restraint stress	No bedding	Predator smells	Tilted cage	No bedding+tilted cage	Predator smells	Light cycle disturbance
	Wet bedding	Tilted cage	No bedding+water	Social stress	Wet bedding		
**Week 2**	No bedding+water	Tilted cage	Predator smells	No bedding	Social stress	Social stress	Restraint stress
	Predator smells	Social stress	Wet bedding	No bedding+tilted cage	Wet bedding		
**Week 3**	Tilted cage	Social stress	Predator smells	Social stress	No bedding	Restraint stress	Light cycle disturbance
	Wet bedding	No bedding+water	No bedding+tilted cage	Wet bedding	No bedding+tilted cage		
